# Clinical Characteristics of Local Recurrent Basal Cell Carcinoma After Surgical Excision: A Retrospective Study of the Patients From a Tertiary Clinical Center

**DOI:** 10.7759/cureus.66668

**Published:** 2024-08-12

**Authors:** Elif Bal Avci, Ayşe Esra Koku Aksu, Dilara Ilhan Erdil, Vefa Aslı Erdemir, Cem Leblebici

**Affiliations:** 1 Department of Dermatology, Bayburt State Hospital, Bayburt, TUR; 2 Department of Dermatology, University of Health Sciences, Istanbul Training and Research Hospital, Istanbul, TUR; 3 Department of Dermatology, Ankara Etlik City Hospital, Ankara, TUR; 4 Department of Dermatology, Medeniyet University, Istanbul, TUR; 5 Department of Pathology, University of Health Sciences, Istanbul Training and Research Hospital, Istanbul, TUR

**Keywords:** surgical excision, skin cancer, recurrence, dermatologic surgery, basal cell carcinoma

## Abstract

Background: Basal cell carcinoma (BCC) is the most frequent form of skin cancer. The etiology of recurrent BCC is multifactorial, and the recurrence rate is variable.

Objective: The aim of the study was to identify the risk factors of local recurrence after surgical excision in primary BCC.

Materials and methods: In our study, 934 patients histopathological diagnosed with BCCs between January 2017 and June 2022 were evaluated retrospectively. Among these, patients who were regularly followed up for at least three years were included in the study. Patients who underwent non-excision treatment were excluded. All the patients who had pathologically confirmed, surgically excised BCCs with safety margins and those with a clinicopathological diagnosis of recurrent BCCs. Demographic and clinical features of 78 patients with non-recurrent primary BCC and 55 patients with local recurrent BCC were compared.

Results: The mean age was 69.7±11.7 years. The gender distribution was M/F:1.3. The time from diagnosis to total surgical excision was 2.3±1.4 months, and the time of recurrence was 27.5±23.3 months. The age of the patients, the time from diagnosis to total excision, the lesion size > 2 cm, and the presence of risk factors (such as radiotherapy, malignancy, and immunosuppression) were higher in the recurrent group than in the non-recurrent group (p < 0.05). Location (high/medium/low-risk area) and the presence of multiple lesions did not differ significantly between the recurrent and non-recurrent groups.

Conclusion: In patients with BCC, recurrence is often detected in the first three years after diagnosis. Our study determined age, lesion size, accompanying risk factors, and the length of time until total excision as risk factors for recurrence in BCC patients. The histological subtype and lesion localization did not differ between the group with and without recurrence.

## Introduction

Basal cell carcinoma (BCC) is a prevalent skin cancer with an increasing incidence worldwide [1]. UV exposure is the most important carcinogenic factor [2]. Other risk factors are male gender, Fitzpatrick types I and II, a personal history of BCC, chronic arsenic exposure, exposure to ionizing radiation, immunosuppression, genetic syndromes, and family history [3]. Treatment options for BCC include surgical excision, Mohs micrographic surgery (MMS), curettage and electrodesiccation, topical agents, photodynamic therapy, cryotherapy, and radiation therapy. Tumor characteristics, such as size, location, and pathology, as well as treatment tolerability, cost, and patient preference influence the selection of treatment. High-risk BCCs include those located in high-risk areas (central face, nose, lips, eyelids, eyebrows, periorbital skin, chin, mandible, ears, preauricular and postauricular areas, temples, hands, feet); tumors ≥20 mm on trunk or limbs; tumors with aggressive pathologic features; perineural involvement; poorly defined border; recurrent; immunosuppression; or site of prior radiation therapy [4]. Surgical excision is generally recommended as first-line therapy for BCC at low risk of recurrence [5]. BCC lesions smaller than 2 cm in diameter, excision with 4-mm clinical margins should result in complete removal in more than 95% of cases [6]. There are no data from randomized trials on the appropriate margin size for high-risk BCC. Postoperative identification of clear margins is critical with standard excision. Follow-up for BCC patients should be evaluated every six to 12 months for the first five years after treatment and then at least once per year [4]. Etiological factors of recurrent BCC are multifactorial, recurrence rate is between 1.2% and 23% [7-10]. Factors influencing the risk of recurrence include the tumor's size, location, histological subtype, and treatment modalities [5].

The aim of this study is to investigate the characteristics of recurrent BCC and identify potential factors that may influence the risk of recurrence. The research will assess the epidemiological, clinical, and histopathological features of recurrent BCC, identify risk factors contributing to recurrence rates, and provide foundational knowledge for developing strategies to prevent recurrences.

## Materials and methods

In our study, 934 patients histopathologically diagnosed with BCCs between January 2017 and June 2022 were evaluated retrospectively. We obtained patients' data from the medical records and surgical pathology database. Among these, patients who were regularly followed up for at least three years were included in the study. Patients who underwent non-excision treatment were excluded. All the patients had pathologically confirmed, surgically excised BCCs with safety margins and those with clinicopathological diagnoses of local recurrent BCCs. Seventy-eight patients with non-recurrent primary BCC and 55 patients with local recurrent BCC of demographic, clinical, and histopathological features were compared.

Patients clinically and dermoscopically evaluated as BCC were diagnosed with punch biopsy. Patients with confirmed histopathology underwent surgical excision with a margin of 4-6 mm, depending on the characteristics of the lesion area. If patients had positive margins, after the initial excision, they underwent re-excision to achieve clear surgical margins. Patients received postoperative follow-up at our clinic every 6 months. Patients were warranted with regards to self-examination and to report for a medical examination if they suspected a skin abnormality around the operation field. In the follow-up, patients with local recurrence suspected clinically and dermoscopically were evaluated hispathologically. Patients diagnosed with local recurrence were treated with surgical excision. The clinical characteristics of patients who were followed up regularly for at least three years and developed no recurrence and who developed local recurrence were compared. The first recurrent lesion was evaluated in patients with multiple recurrences. The research protocol received approval from the Research Ethics Committee of the Istanbul Training and Research Hospital (IRB number: 05.08.2022/248).

Statistical analysis of the data was performed using the program IBM SPSS version 28.0 (IBM Corp., Armonk, NY). Descriptive statistics, including mean, standard deviation, median, minimum, maximum, frequency, and ratio values, were utilized in the analysis of the data. The distribution of variables was assessed using the Kolmogorov-Smirnov test. The Mann-Whitney U test was employed for the analysis of quantitative independent variables. For the analysis of independent variables, the chi-square test was used, and in cases where the chi-square test conditions were not met, the Fisher's exact test was employed.

## Results

A total of 133 BCC patients were included in the study. The mean age was 69.7 (28-90 SD:11.7). The gender distribution was M/F:1.3. Fifteen (12.8%) of the BCC patients had risk factors such as immunosuppression, radiotherapy, and malignancy. Actinic keratosis was observed in 16 patients (12%), SCC in 11 patients (8.3%), and melanoma was additionally observed in one patient (0.8%) (Table 1). One hundred fifteen tumors (86.5%) were under 2 cm in diameter. In 77 patients (57.9%), most tumors were located in high-risk area and 65 patients (48.9%) had a single lesion, and 68 patients (51.1%) had multiple lesions. When unclassifiable BCCs were not considered, the most common primary tumor subtypes were mixed (n:36, 27%) and nodular (n:33, 24.8%) histological subtypes. Similarly, mixed (n:20, 15%) and nodular (n:17, 12.8%) types were the most common in recurrent tumors. When the histological subtypes of the recurrent and non-recurrent groups were compared, due to many subtypes no significant results were obtained (Table 2). The time from diagnosis to total surgical excision was 2.3±1.4 months, and the time of recurrence was 27.5±23.3 months. The recurrence rate in our clinic in BCC patients followed for at least three years was 5.88%. The age of the patients, the time from diagnosis to total excision, the lesion size > 2 cm and the presence of risk factors (such as radiotherapy, malignancy, immunosuppression) were higher in the recurrent group than in the non-recurrent group (p <0.05). Location (high/medium/low-risk area) and the presence of multiple lesions did not differ significantly between the recurrent and non-recurrent group (Table 3).

**Table 1:** Clinical and demographic characteristics of patients

		Mean ±SD	
Age		69.7±11.7	
		Number	Percentage
Gender	Male	76	57.1
	Female	57	42.9
Concomitant cutaneous malignancy	Absent	104	78.2
	Actinic Keratosis	16	12.0
	SCC	11	8.3
	Melanoma	1	0.8
Risk factors	Absent	116	87.2
	Malignancy	5	3.8
	Radiotherapy	5	3.8
	Immunosuppression	6	4.5
	Gorlin syndrome	1	0.8

**Table 2:** Evaluation of BCC location, lesion size, and histological subtypes of patients BCC - Basal cell carcinoma

		Number	Percentage
Primary lesion size (cm)	<2	115	86.5
	>2	18	13.5
Primary lesion location	H	77	57.9
	L	17	12.8
	M	39	29.3
Multiple lesion	No	65	48.9
	Yes	68	51.1
Primary BCC histological subtype	Nodular	33	24.8
	Superficial	6	4.5
	Morpheaform	2	1.5
	Micronodular	5	3.7
	Infiltrative	3	2.2
	Basosquamous	2	1.5
	Fibroepithelial	3	2.2
	Infundibulocystic	1	0.7
	Adenoid	5	3.7
	Mixed	36	27
	Unclassified	37	27.8
Recurrence histological subtype	Nodular	17	12.8
	Superficial	1	0.8
	Micronodular	1	0.8
	İnfiltrative	4	3.0
	Mixed	20	15.1
	Unclassified	12	9.0

**Table 3:** Comparison of clinical features of the recurrence group and the non-recurrence group m: Mann-Whitney U test, X²: Chi-square test

			Recurrence (-)		Recurrence (+)		p	z value	x² value
			Avg.±SD		Avg.±SD				
Age			66.7±12.3		74.1±9.2		0.000^m^	-3.623	-
The time from diagnosis to total surgical excision (month)			2.0±1.6		2.6±1.1		0.000^m^	-3.927	-
			Number	Percentage	Number	Percentage			
Gender		Male	46	59.0	30	54.5	0.611^X²^	-	0.258
		Female	32	41.0	25	45.5			
Primary lesion size (cm)		< 2	72	92.3	43	78.2	0.019^X²^	-	5.500
		> 2	6	7.7	12	21.8			
Primary lesion location		H	46	59.0	31	56.4	0.573^X²^	-	1.114
		L	8	10.3	9	16.4			
		M	24	30.8	15	27.3			
Multiple lesion		No	35	44.9	30	54.5	0.272^X²^	-	1.208
		Yes	43	55.1	25	45.5			
Concomitant cutaneous malignancy		Absent	59	75.6	45	81.8	0.396^X²^	-	0.722
		Actinic Keratosis	13	16.7	3	5.5			
		SCC	4	5.1	7	12.7			
		Melanoma	1	1.3	0	0.0			
Risk factor		Absent	72	92.3	44	80.0	0.036^X²^	-	4.383
		Malignancy	2	2.6	3	5.5			
		Radiotherapy	0	0.0	5	9.1			
		Immunosuppression	4	5.1	2	3.6			
		Gorlin Syndrome	0	0.0	1	1.8			

## Discussion

BCC is the most commonly observed cancer worldwide [11,12]. The incidence of BCC is steadily increasing, emphasizing the growing importance of early diagnosis and effective treatment. BCC generally follows an indolent course, and there are various treatment methods available. However, despite these treatment options, recurrence rates can vary significantly depending on the chosen method. MMS is the recommended treatment for high-risk tumors. In our study, cases that developed within the boundaries of the scar and were histopathologically confirmed were considered a recurrence definition. Various studies have identified different risk factors for recurrence. Recurrence rates for BCC are between 1.2% and 23% [7-10]. Recurrence rate varies widely according to treatment modality and risk factors. In primary BCC, the recurrence risk is 1.5%, while the rate increases to 13.2% when BCC has been previously treated [13]. Risk factors for recurrence include the patient's age, non-primary tumors, cases treated before MMS, those requiring multiple stages of MMS, BCC left for secondary healing after surgery, incomplete excision, margin positivity (deep and lateral margins), tumor location (head/neck), tumoral depth (beyond fat), histopathological subtype (infiltrative and micronodular), chosen treatment modality (curettage or PDT) having a higher risk of recurrence compared with excision and radiotherapy). Low-risk situations for recurrence are early diagnosis age and early stage, excision repairing with a local skin flap [7-9,13-21].

In our study, the patients had an average age of 69.7 years, and the M/F ratio (1.3) was determined to be consistent with the literature [8,16,22,23]. After histopathological verification through biopsy, total excision was conducted within a two-month period. Recurrence occurred on an average of 27.5 months later. The average reported time for the development of recurrence is reported as between 4.5 and 31.2 months [7,14,16,19,23]. The determination of an average recurrence duration of over two years in our study emphasizes the importance of patient follow-up. Even if the frequency of patient follow-ups decreases, patients should not be discontinued from follow-up since recurrence can occur relatively late.

The extended time from diagnosis to excision has been identified as a risk factor in our study. There is insufficient data in the literature on this matter, and further studies may reveal the relationship between this aspect and histopathological aggressiveness, considering that BCC is a relatively slow-progressing tumor.

In our study, age was identified as one of the risk factors for recurrence. A younger age during the diagnosis is reported as a protective factor, while advanced age has been previously indicated as a risk factor for recurrence [14]. According to Velazquez et al., there is a 24% increased risk of recurrence for every 10 years of age increment [16]. However, various studies also exist where age is not identified as a risk factor for recurrence [18,24]. Another study found a higher recurrence rate in younger patients [23].

The presence of a lesion larger than 2 cm is found to be a risk factor for recurrence which has been previously identified as one of the reported risk factors [14]. However, after the application of MMS, size is not listed as a risk factor [13]. Therefore, when MMS is performed, this risk becomes less significant, and there is no statistically significant association with recurrence [16]. Another study involving patients treated with MMS also did not find size to be a significant risk factor [25]. This demonstrates the variability of risk factors based on the selected treatment modality.

In our study, the presence of another malignancy has been identified as a recurrence risk factor. For patients with BCC, the risk of having a different malignancy has increased, such as other skin malignancies or respiratory cancers [26,27]. Furthermore, individuals with multiple non-melanoma skin cancers at the initial occurrence also demonstrated an increased risk of recurrence for BCC [28].

The history of radiotherapy has also been identified as a risk factor in our study; however, it is unknown whether the exact localization of radiotherapy coincides with the region affected by BCC. According to a study conducted on patients with a history of radiation therapy due to childhood cancers, radiation doses to the skin exceeding 1 Gy are associated with an increased risk of BCC [29]. Immunosuppression has also been identified as a risk factor for recurrent BCC, with a 14.5% incidence of BCC development in patients after renal transplantation [30]. In individuals with a history of HIV, a higher risk of recurrence has been observed [28].

Lesion location (high or low risk) has not been identified as a risk factor for recurrence in our study. This is variable in the literature, and some studies do identify it as a risk factor [13,14,18]. Nevertheless, it remains one of the important criteria when choosing the treatment modality.

The most significant limitation of our study is that it was conducted in a single center retrospectively. The second limitation is that none of our patients underwent MMS, since it is not practiced in our center. Therefore, considering that some risk factors for recurrence may change when MMS is performed, the applicability of the identified risk factors in our study is limited to patients who underwent standard total excision only.

## Conclusions

BCC is the most common form of skin cancer, known for its slow progression and local invasiveness. Despite being highly treatable, BCC presents a significant clinical challenge due to its high recurrence rates. Although recent advancements in the treatment of BCC have been significant, recurrence remains a substantial issue, necessitating a deeper understanding of the mechanisms behind recurrence and the development of new treatment strategies that could potentially reduce the risk of recurrence.

Despite Mohs surgery being the gold standard, especially in high-risk groups, it is still not an easily accessible treatment method in most centers. Surgical excision is an easy and cost-effective treatment method, and when performed with a safe margin, recurrence is seen at a low rate. Our study determined age, lesion size, accompanying risk factors, and the length of time until total excision as risk factors for recurrence in BCC patients. The histological subtype and lesion localization did not differ between the group with and without recurrence.
